# Synthetic tissues lack the fidelity for the use in burn care simulators

**DOI:** 10.1038/s41598-022-25234-x

**Published:** 2022-12-10

**Authors:** Vanessa Hannay, F. N. U. Rahul, Kartik Josyula, Uwe Kruger, Samara Gallagher, Sangrock Lee, Hanglin Ye, Basiel Makled, Conner Parsey, Jack Norfleet, Suvranu De

**Affiliations:** 1 Department of Biomedical Engineering, Rensselaer Polytechnic Institute, Troy, NY USA; 2 Department of Mechanical, Aerospace, and Nuclear Engineering, Rensselaer Polytechnic Institute, Troy, NY USA; 3 Center for Modeling, Simulation, and Imaging in Medicine, Rensselaer Polytechnic Institute, Troy, NY USA; 4 U.S. Army Futures Command, Combat Capabilities Development Command Soldier Center STTC, Orlando, FL USA

**Keywords:** Machine learning, Biomedical materials

## Abstract

This work compares the mechanical response of synthetic tissues used in burn care simulators from ten different manufacturers with that of ex vivo full thickness burned porcine skin as a surrogate for human skin tissues. This is of high practical importance since incorrect mechanical properties of synthetic tissues may introduce a negative bias during training due to the inaccurate haptic feedback from burn care simulator. A negative training may result in inadequately performed procedures, such as in escharotomy, which may lead to muscle necrosis endangering life and limb. Accurate haptic feedback in physical simulators is necessary to improve the practical training of non-expert providers for pre-deployment/pre-hospital burn care. With the U.S. Army’s emerging doctrine of prolonged field care, non-expert providers must be trained to perform even invasive burn care surgical procedures when indicated. The comparison reported in this article is based on the ultimate tensile stress, ultimate tensile strain, and toughness that are measured at strain rates relevant to skin surgery. A multivariate analysis using logistic regression reveals significant differences in the mechanical properties of the synthetic and the porcine skin tissues. The synthetic and porcine skin tissues show a similar rate dependent behavior. The findings of this study are expected to guide the development of high-fidelity burn care simulators for the pre-deployment/pre-hospital burn care provider education.

## Introduction

Burns are some of the most common injuries in both civilian and combat scenarios. Acute burn injury occurs in approximately 5 to 20% of combat casualties^1^. Adequate initial care within the first hour of post-burn injury is known to impact the long-term recovery of the patient. However, the initial care is often performed outside of a dedicated burn care center due to lack of immediate accessibility and transferability to the centers^2^. When the burn injuries occur in a rural/austere environment in civilian and combat situations, the burn patients might be prevented from transfer to the burn centers for several days. A delay in performing burn care including clinically indicated surgical interventions, or inadequately performed burn care procedures, may cause complications such as muscle necrosis and in severe cases, limb amputation^3^. The U.S. Army’s medical doctrine is also evolving to include prolonged field care (PFC), and with burns being a common injury among soldiers, non-expert providers must be adequately trained to perform burn care, including invasive surgical procedures, as and when the situation demands. Existing medical trainers, however, fall short in providing necessary fidelity for adequate training^4^.

Moulages on low-fidelity mannequins are often used in the practicum portion of the American Burn Association's Advanced Burn Life Support (ABLS) course to provide the visual cues and appearance of a burn injury in order to gain the practical experience of treating burn injuries^5–7^. The ABLS course is considered the standard for training civilian and military medical personnel in burn care. Moulages have also been created to mimic the acute burn scenario to enable invasive burn care procedures, e.g., escharotomy, to be performed in a resource-limited environment^8–12^. High-fidelity burn care simulators focus on creating physiological cues and team-building environments^6,13^. However, these simulators lack the mechanical response and haptic feedback necessary for the trainees to learn prolonged burn care procedures. A recent study has shown a significant difference in the mechanical behavior of synthetic materials used in a variety of medical trainers and human cadaveric pleura tissues^4^. This disparity in mechanical behavior may introduce negative training by requiring vastly different forces and energies to complete intrusive, yet delicate medical procedures on medical trainers. For example, a negative training of an escharotomy procedure may result in practitioners damaging underlying vasculature and inflicting infection-prone trauma in live patients. Hence, it is imperative first to evaluate the mechanical response of synthetic tissues used in physical simulators with respect to that of the burned skin tissues. Such analysis may guide the future development of high-fidelity burn care simulators.

The mechanical behavior of various synthetic materials has been studied as a surrogate to human skin tissue. However, the mechanical response of human skin is considered more complex than that of rubber since the skin has a layered, orthotropic, and heterogeneous structure compared to rubber^14^. Porcine skin is often used as a surrogate of human skin to evaluate the mechanical behavior of synthetic tissues. In uniaxial tensile tests, the strain rate sensitivity and hardening behavior of rubber and pig skin are found to be different^15^. It is observed that the tensile strength of synthetic chamois is four times less than that of pig skin^16^. Different compositions of elastomer-based skin surrogates are shown to accurately describe the nonlinear stress–stretch behavior, the elastic modulus at high and low strains, and the fracture strengths of the human skin at different anatomical locations^17^. However, these studies are limited to unburned skin tissue, which is known to exhibit stiffer stress–strain response compared to burned skin tissues^18^. In addition, the mechanical characteristics of commercially available synthetic tissues as a surrogate of the full thickness burned skin tissues have not been reported.

This work characterizes the mechanical properties of ex vivo full thickness burned porcine skin tissues and synthetic phantoms from various manufacturers using uniaxial tensile tests. The overall similarities between porcine and human skin make pigs the most favorable surrogate for humans in burn experiments^19^. Controlled burn experiments on fresh ex vivo porcine skin tissues are performed to inflict full thickness burns. Rate effects of the synthetic tissues are also studied by testing the samples under different loading rates relevant to skin surgery. In addition to the stress–strain response, ultimate tensile stress, ultimate tensile strain, and toughness are obtained for each sample. A multivariate statistical analysis^20^ is performed using logistic regression^21^ to evaluate the fidelity of synthetic tissues by comparing their mechanical properties with those of ex vivo full thickness burned tissues characterized at various loading rates. The rate dependent behavior is analyzed using a multiclass multivariate analysis with logistic regression and kernel Fisher discriminant analysis^22^. The leave-one-out cross validation is applied to assess the classification analysis.

The remainder of this paper is organized as follows. In “Methods and materials” section, the details of the sample preparation, uniaxial tensile testing protocol, including the data collection process, and the statistical analysis technique are described. “Results and discussion” section discusses the results, followed by conclusion and future directions in “Conclusion” section. The confusion matrices from the classification analysis are listed in Appendix A in supplementary information.

## Methods and materials

This work aims to evaluate the fidelity of different types of synthetic tissues used in physical simulators. The synthetic tissues and ex vivo full thickness burned porcine skin tissues are characterized using mechanical properties such as ultimate tensile (UT) stress, UT strain, and toughness. Multivariate statistical analysis is performed to evaluate the fidelity of synthetic tissues compared to burned porcine skin tissues based on the measured mechanical properties. The sample preparation, uniaxial tensile testing, and statistical analysis are described in  “Sample preparation, Uniaxial tensile testing and Statistical analysis” sections respectively.

### Sample preparation

A porcine model was used to obtain the ex vivo full thickness burned skin samples. Locally sourced fresh porcine abdominal skin with subcutaneous and underlying muscles were used in this study. The skin was separated from the subcutaneous and underlying muscles using a scalpel. The specimens were kept hydrated at room temperature in 1X phosphate-buffered saline solution before inflicting full thickness burns using a commercial griller (Cuisinart® GR-300WS Griddler Elite Grill, Conair Corporation, NJ). The specimens were then burned at 450°F (232.2°C) for 30 s. This burn condition is chosen as a surrogate of burn depth, which is known to result in full thickness burn^23^. The burned specimens were punched into a standard dog-bone-shape using an ASTM D638 Type V die after cooling down to the room temperature, confirmed using an infrared camera (InfraCam, FLIR, OR). This was done to avoid shrinkage of skin tissue during the contact heating process. The synthetic tissue samples were also processed into dog-bone-shape specimens following the same ASTM standards as the porcine tissues. A list of commercial synthetic simulated skin products from various manufacturers and the number of dog-bone specimens used in this study are provided in Table 1. The total number of samples of burned porcine skin tissue is also listed in Table 1. The mean and standard deviation of the sample thickness for the synthetic tissues and burned porcine skin tissue is provided in Table 1.

**Table 1:** Tissue groups and number of samples for the three loading rates.

Tissue group number	Skin tissue description*	Number of samples			Sample thickness (mm)
		0.3 mm/s	2 mm/s	8 mm/s	
1	Skin SIM 1	15	15	15	1.5 ± 0.2
2	Neck Skin 1	15	15	15	2.3 ± 0.3
3	Neck Skin 2 (Thick)	15	15	15	4.3 ± 0.5
4	Chest Skin	15	15	15	2.8 ± 0.5
5	Skin SIM 2	15	15	15	1.0 ± 0.1
6	Skin Crest	15	15	15	1.0 ± 0.1
7	Airway Skin	15	15	15	1.7 ± 0.1
8	Skin SIM 3	15	15	15	2.9 ± 0.2
9	Neck Skin 3	15	15	15	1.8 ± 0.5
10	Neck Skin 4 (Thin)	15	15	15	2.1 ± 0.4
11	Burned Porcine Skin	17	24	10	2.7 ± 0.3

*This research is a comparison of material properties and does not constitute an endorsement nor a criticism of any commercial product.

### Uniaxial tensile testing

The uniaxial tensile tests were performed on an Instron® MTS system (INSTRON, MA). The dog-bone specimens in each group (Table 1) were tested at three different loading rates, *i.e.,* 0.3 mm/s, 2 mm/s, and 8 mm/s. While the 0.3 mm/s rate corresponds to a quasi-static loading condition, the other two are chosen to be consistent with the cutting speed during skin surgery^24^. The specimens were uniaxially stretched until rupture. The force and displacement curves were recorded for each test. The nominal stress (${\sigma }_{N}$) is obtained by dividing the applied force $F$ by the initial cross-sectional area ${A}_{0}$ of the specimen normal to the loading direction, i.e., ${\sigma }_{N}=F/{A}_{0}$. The nominal strain (${\varepsilon }_{N}$) is computed as the ratio of measured displacement $\Delta L$ and initial sample length ${L}_{0}$, i.e., ${\varepsilon }_{N}=\Delta L/{L}_{0}$. The ultimate tensile (UT) stress is defined as the maximum value of the nominal stress prior to rupture, and the UT strain is the corresponding nominal strain value. Toughness is calculated as the area under the nominal stress–strain curve up to the point of rupture, indicating the energy required to break the tissue specimen. A data set comprised of UT stress, UT strain, and toughness is compiled for each sample.

### Statistical analysis

Multivariate statistical analysis^20^ is performed to test the null hypothesis, ${H}_{0}$: synthetic tissue has the same mechanical properties as the full thickness burned tissue, against the alternative hypothesis, ${H}_{\mathrm{a}}$: synthetic and full thickness burned tissues have different mechanical properties. Univariate analysis alone may not offer an insight into the significance of the differences between the mean or median of mechanical properties of porcine skin and synthetic tissues.

Logistic regression models^21^ are trained to separate porcine skin tissues from each of the ten synthetic tissues using all three properties for samples at three loading rates individually as well as combined. The logistic regression model^21^ and the kernel Fisher discriminant analysis^22^ are used to distinguish the samples loaded at the three different loading rates for the burned porcine skin tissue and each of the ten synthetic tissues. A leave-one-out cross validation (LOOCV)^25^ is performed, which involves splitting the sample set into a training set containing all but one observation and a validation set that includes the observation left out. Since the excluded observation is not used for training, the misclassification error provides an independent estimate for the accuracy of the classifier. Unlike the validation set approach, which randomly splits the dataset into mutually exclusive training and validation sets, LOOCV does not lead to variability in the test error that may arise due to random partitioning of the dataset and an insufficient number of samples in the training and validation sets. The performance of the classification analyses is evaluated using accuracy, sensitivity, specificity, F1 score, and area under the receiver operating characteristic curve (AUC-ROC), and multiclass metrics of Fowlkes-Mallows index (FMI)^26^, adjusted Rand index (ARI)^27^, and Matthews correlation coefficient (MCC)^28^ that are computed using a confusion matrix. The contribution of each of the three properties towards the classification accuracy is calculated using factor analysis.

## Results and discussion

Uniaxial tensile tests are performed on full thickness burned porcine skin tissue and 10 synthetic tissues used in commercial applications at three loading rates, i.e., 0.3 mm/s, 2 mm/s, and 8 mm/s. The ultimate tensile (UT) stress, the UT strain, and the toughness are calculated using the force–displacement data from the tensile tests. The results are presented in “Results” section and the mechanical behavior of the synthetic tissues and burned porcine skin tissue based on the results is discussed in “Discussion” section.

### Results

For each of the 10 synthetic tissues and the full thickness burned porcine tissue, the outlier samples are identified for each loading rate as the samples with any of the three mechanical properties beyond three times the interquartile range of the corresponding distribution of the property. These outlier samples are not used for the multivariate statistical analysis. The sample sizes for each of the 10 synthetic tissues and the porcine tissue that are used for the statistical analysis at each loading rate are given in Table 2.

**Table 2:** Tissue groups and number of samples for the three loading rates after removal of outliers.

Tissue group number	Skin tissue description	Number of samples		
		0.3 mm/s	2 mm/s	8 mm/s
1	Skin SIM 1	15	15	15
2	Neck Skin 1	15	15	15
3	Neck Skin 2 (Thick)	14	15	15
4	Chest Skin	15	15	14
5	Skin SIM 2	15	14	15
6	Skin Crest	15	15	15
7	Airway Skin	15	15	15
8	Skin SIM 3	15	15	14
9	Neck Skin 3	15	15	15
10	Neck Skin 4 (Thin)	15	15	13
11	Burned Porcine Skin	17	24	10

**Figure 1:**
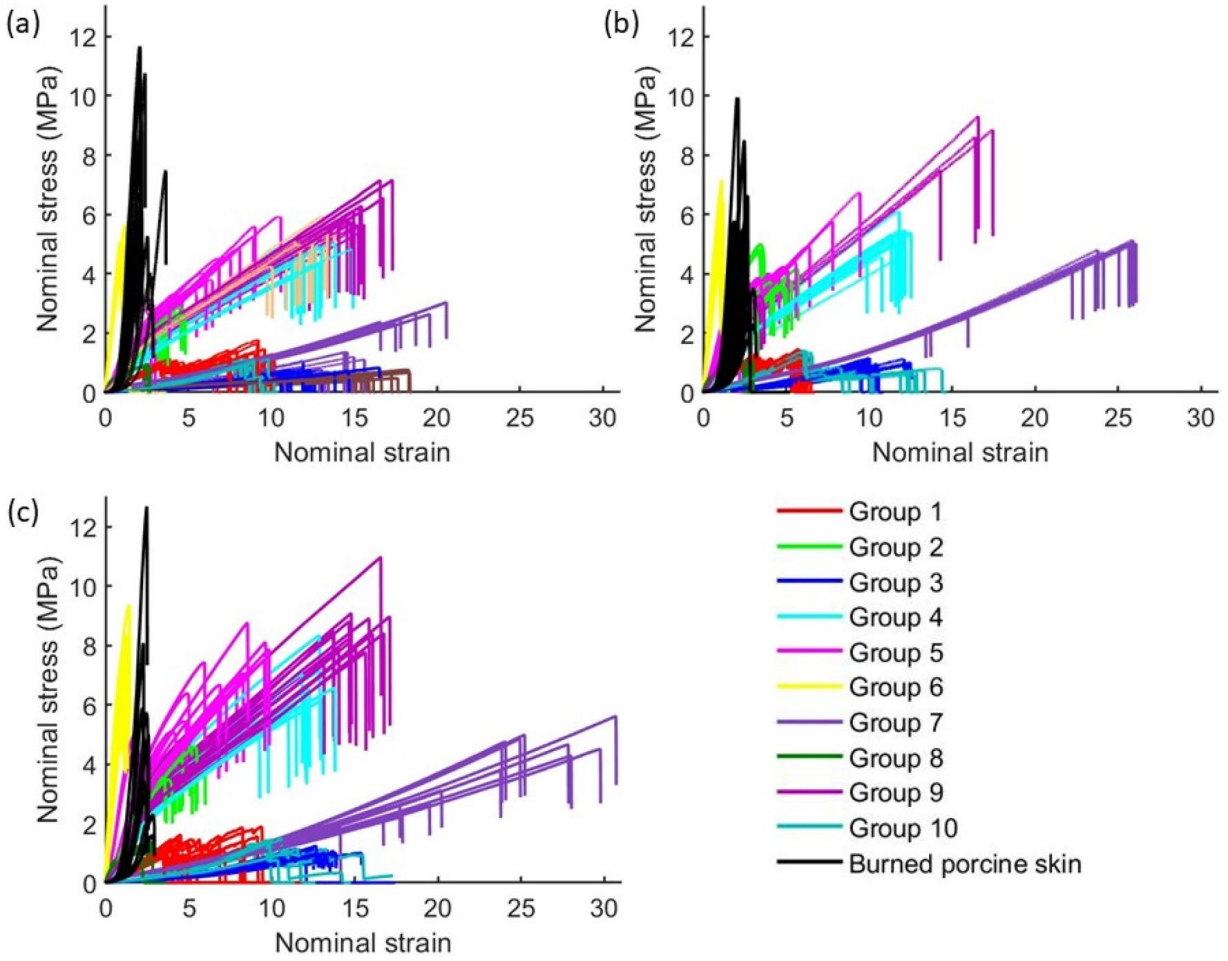
Nominal stress–strain curves of synthetic tissue samples and full thickness burned porcine skin tissue samples loaded at (**a**) 0.3 mm/s, (**b**) 2 mm/s, and (**c**) 8 mm/s. Curves are shown until the rupture point.

**Figure 2:**
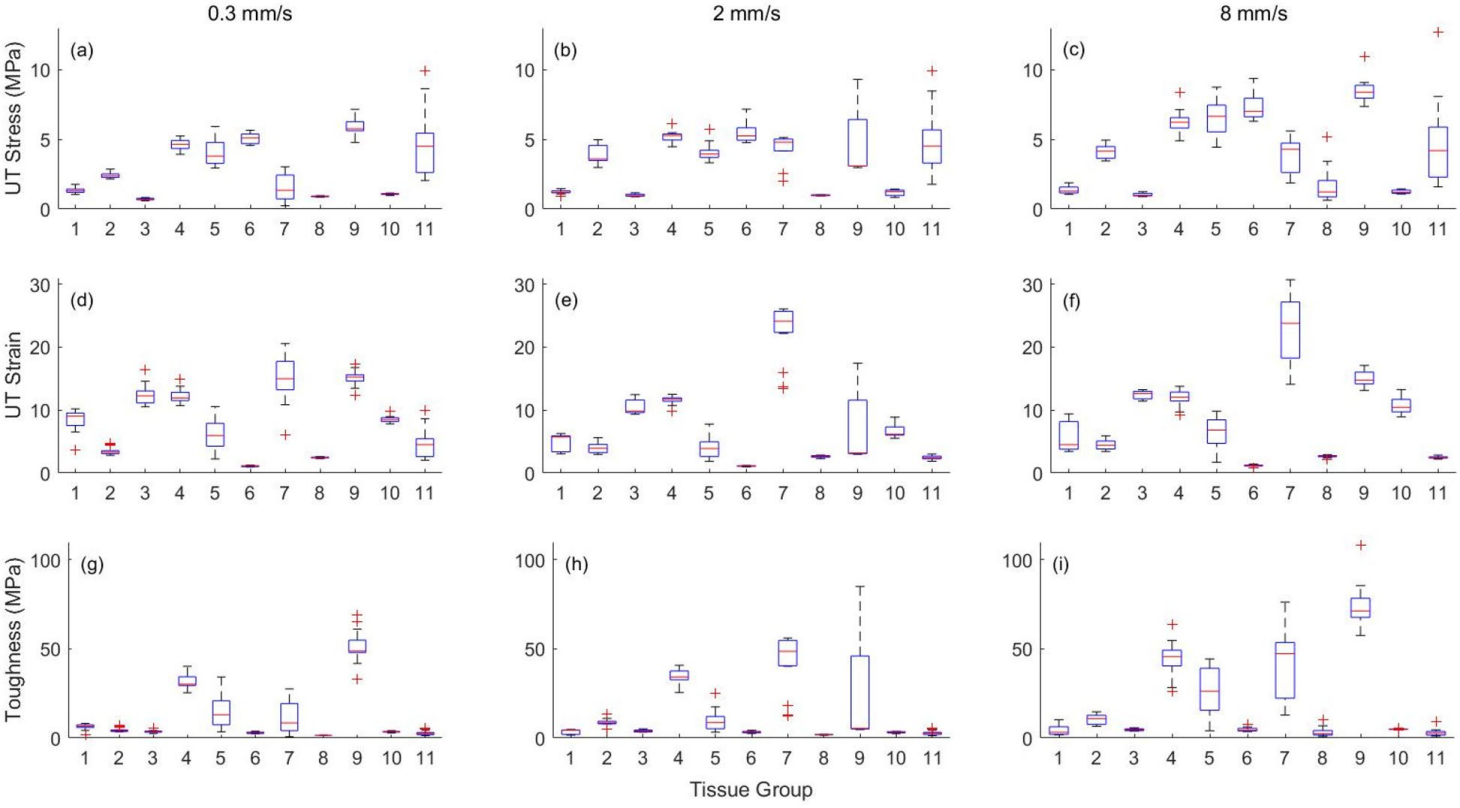
Boxplots of (**a**)–(**c**) ultimate tensile stress (UT Stress), (**d**)–(**f**) ultimate tensile strain (UT Strain), and (**g**)–(**i**) toughness of synthetic (tissue groups 1–10) and full thickness burned porcine skin (tissue group 11) tissue samples loaded at 0.3 mm/s, 2 mm/s, and 8 mm/s.

The nominal stress–strain plot for loading of the synthetic tissues and full thickness burned porcine skin tissue at the three loading rates is shown in Fig. 1. The box plots of the three mechanical properties of the 10 synthetic tissues and the porcine skin tissue are given in Fig. 2 for the three loading rates.

From Fig. 2, the median and variance of the UT stress of synthetic tissues are observed to be different from the porcine skin tissue. At all three loading rates, the porcine skin tissue has larger UT stress with larger variance than the synthetic tissues. The UT strain of the synthetic tissues is more than that of the burned skin tissue at the three loading rates, except for tissue groups 6 and 8. Further, the burned skin tissue has a smaller variance for UT strain compared to synthetic tissue samples at the loading rates of 2 mm/s and 8 mm/s, which are relevant for skin surgery. The toughness of the synthetic tissues is comparable to that of the burned skin tissue samples at all loading rates, except for tissue groups 4, 5, 7, and 9. This indicates that it requires similar energy for the burned skin and synthetic samples to rupture.

The binary classification using logistic regression is carried out to differentiate porcine skin from each of the 10 synthetic tissues based on three mechanical properties. All of the performance metrics, i.e., accuracy, sensitivity, specificity, F1-score, and AUC-ROC, and MCC, computed from the confusion matrix obtained from LOOCV for each of the three loading rates are found to be 100%. For the binary classification of samples from all loading rates combined, the performance metrics are given in Table 3. The confusion matrices for the classifications are provided in supplementary information (Appendix A).

**Table 3:** Performance metrics for the classification of full thickness burned porcine skin and synthetic tissue samples loaded at all three rates combined.

Porcine versus synthetics	Accuracy	Sensitivity	Specificity	F1-score	AUC-ROC	MCC
1	1.0	1.0	1.0	1.0	1.0	1.0
2	1.0	1.0	1.0	1.0	1.0	1.0
3	1.0	1.0	1.0	1.0	1.0	1.0
4	1.0	1.0	1.0	1.0	1.0	1.0
5	0.99	0.98	1.0	0.99	0.99	0.98
6	1.0	1.0	1.0	1.0	1.0	1.0
7	0.99	1.0	0.98	0.99	0.99	0.98
8	1.0	1.0	1.0	1.0	1.0	1.0
9	1.0	1.0	1.0	1.0	1.0	1.0
10	1.0	1.0	1.0	1.0	1.0	1.0

The performance metrics for all the binary classification analyses indicate that each of the 10 synthetic tissue types are statistically distinguishable from the porcine skin tissue for three loading rates. This implies that the mechanical characteristics of the two tissue types are significantly different. Hence, the null hypothesis is rejected in favor of the alternative hypothesis.

**Figure 3:**
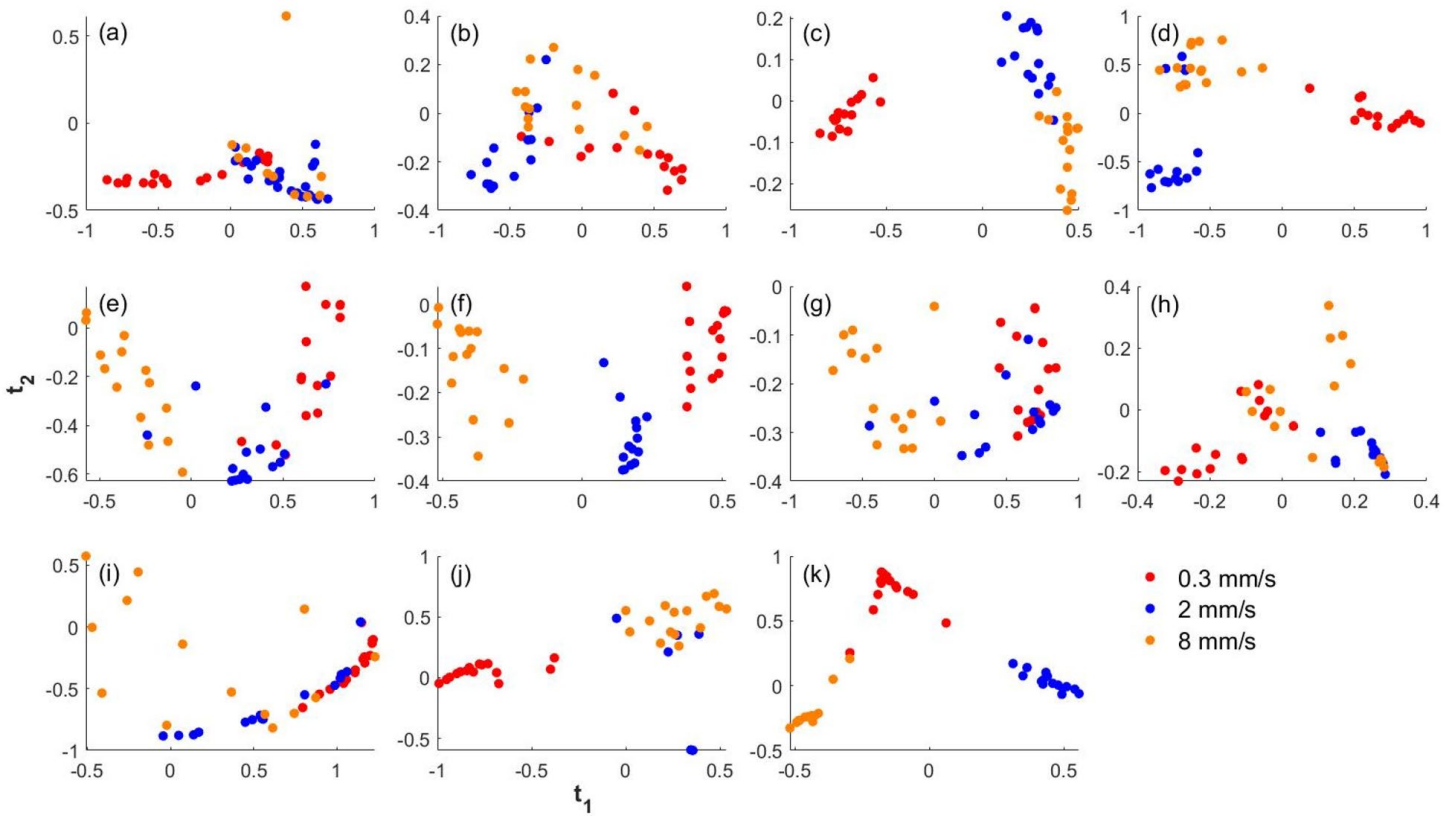
The kFDA scores of the samples of the (**a**) burned porcine skin tissue and the (**b**)–(**k**) 10 synthetic tissues loaded at the three rates along the two projections (t_1_ and t_2_) calculated from the kFDA based classification analysis.

The rate dependence of the burned porcine skin and the ten synthetic tissues is described by the multiclass classification of the samples into the three loading rate groups based on the three material properties. The multiclass classification is performed using logistic regression model and kernel Fisher discriminant analysis (kFDA). The performance metrics of the classification analyses using both the methods for each tissue type are given in Table 4. The clusters of the kFDA scores of the samples for each tissue type along the two projections calculated from the kFDA based classification analysis is shown in Fig. 3. The accuracy of the multiclass classification is > 70% for all tissue types using at least one of the methods, except for the synthetic tissue groups, 1 and 6 whose accuracy is 60–70% using both methods. From Fig. 3, the loading rate of 0.3 mm/s pertaining to the static case is clearly distinguishable from the loading rates of 2 and 8 mm/s, which are relevant to the skin surgery. This is confirmed by the binary classification of the samples of each tissue type which distinguishes the static rate from the rates relevant to surgery using the logistic regression method. The performance metrics of this classification analyses is given in Table 5. The accuracy of the binary classification is > 80% for all tissue types, except for synthetic tissue groups 6 and 10, whose accuracy is > 60%. Hence, the burned porcine skin tissue and the 10 synthetic tissues show rate dependent behavior with a clear separation of samples loaded at static rate (0.3 mm/s) and those loaded at surgical rates (2 and 8 mm/s). The confusion matrices for the classifications are provided in supplementary information (Appendix A).

**Table 4:** Performance metrics for the multiclass classification of the burned porcine tissue and the synthetic tissue samples into the three loading rate classes.

Tissue group number	Logistic regression				Kernel FDA			
	Accuracy	MCC	FMI	ARI	Accuracy	MCC	FMI	ARI
1	0.62	0.44	0.46	0.21	0.64	0.49	0.51	0.26
2	0.78	0.67	0.68	0.53	0.87	0.80	0.77	0.66
3	0.89	0.84	0.80	0.70	0.86	0.81	0.74	0.61
4	0.86	0.80	0.74	0.62	0.75	0.66	0.60	0.38
5	0.95	0.93	0.91	0.87	0.89	0.84	0.78	0.67
6	0.62	0.44	0.61	0.42	0.64	0.49	0.59	0.36
7	0.73	0.62	0.60	0.40	0.67	0.51	0.55	0.33
8	0.86	0.80	0.77	0.67	0.64	0.49	0.52	0.23
9	0.91	0.88	0.84	0.76	0.91	0.88	0.84	0.76
10	0.79	0.69	0.63	0.46	0.91	0.86	0.82	0.73
11	0.61	0.35	0.52	0.15	0.71	0.56	0.63	0.28

**Table 5:** Performance metrics for the binary classification of the burned porcine tissue and the synthetic tissue samples into two classes of static (0.3 mm/s) and surgical (2 and 8 mm/s) strain rates.

Tissue group number	Accuracy	Sensitivity	Specificity	F1-score	AUC-ROC	MCC
1	0.82	0.73	0.87	0.73	0.80	0.60
2	1.0	1.0	1.0	1.0	1.0	1.0
3	1.0	1.0	1.0	1.0	1.0	1.0
4	0.91	0.87	0.93	0.87	0.90	0.80
5	0.98	0.93	1.0	0.97	0.97	0.95
6	0.67	0.53	0.73	0.52	0.63	0.26
7	0.80	0.67	0.87	0.69	0.77	0.54
8	1.0	1.0	1.0	1.0	1.0	1.0
9	1.0	1.0	1.0	1.0	1.0	1.0
10	0.65	0.40	0.79	0.44	0.59	0.20
11	0.82	0.71	0.88	0.73	0.79	0.60

### Discussion

The focus of the present study is to compare the mechanical behavior of ten synthetic skin tissues from various manufacturers with that of the full thickness burned porcine skin tissue using mechanical properties calculated from uniaxial tensile tests on the tissues. These mechanical properties, e.g., ultimate tensile stress and toughness, are standard parameters to describe the mechanical behavior of the burned skin during treatment. These properties have been used in literature to characterize the mechanical response of skin tissue for skin surgery applications^4,17^. The stress–strain curve of synthetic tissue samples in Fig. 1 differ significantly from the characteristic J-shaped curves of the biological skin tissues^29^. At all loading rates, the full thickness burned porcine skin tissue shows deformation behavior that gradually progresses from linear at small strain (phase 1) to nonlinear for medium strains (phase 2) and again linear at large finite strains (phase 3) until ultimate tensile stress is reached. The characteristic three-phase deformation behavior of skin tissue is a manifestation of morphological changes in collagen fiber, which goes from a woven rhombic-shaped pattern at lower strain to a highly aligned state at high strain while interacting with the hydrated matrix^29^. The three-phase stress–strain behavior is absent from all of the synthetic tissues. Similar differences in stress–strain behavior were found between synthetic tissues and human pleura tissue which were suggested to induce negative training in the practitioner and cause harm to patients^4^. Unlike the porcine skin tissues, the stress–strain response of synthetic tissues shows hardening behavior with increasing loading rate. The ultimate tensile (UT) stress and UT strain are significantly different between each of the ten synthetic tissues and the full thickness burned porcine skin tissue. The toughness is similar between the burned porcine tissue and most of the synthetic tissues. These discrepancies in the mechanical behavior are reflected in the statistical analysis as well. These significant differences are also consistent with those observed in the critical force to rupture between synthetic tissues and burned porcine skin tissue during incision and cutting experiments^30^. The differences in mechanical behavior between the synthetic and burned porcine tissues would affect the haptic feedback to the trainees and the haptic cues learned by them using the physical simulators. The lack of haptic cues is a drawback in the practicum portion of the Advanced Burn Life Support course, which is a standard for learning primary treatment of burn injuries^6^.

**Figure 4:**
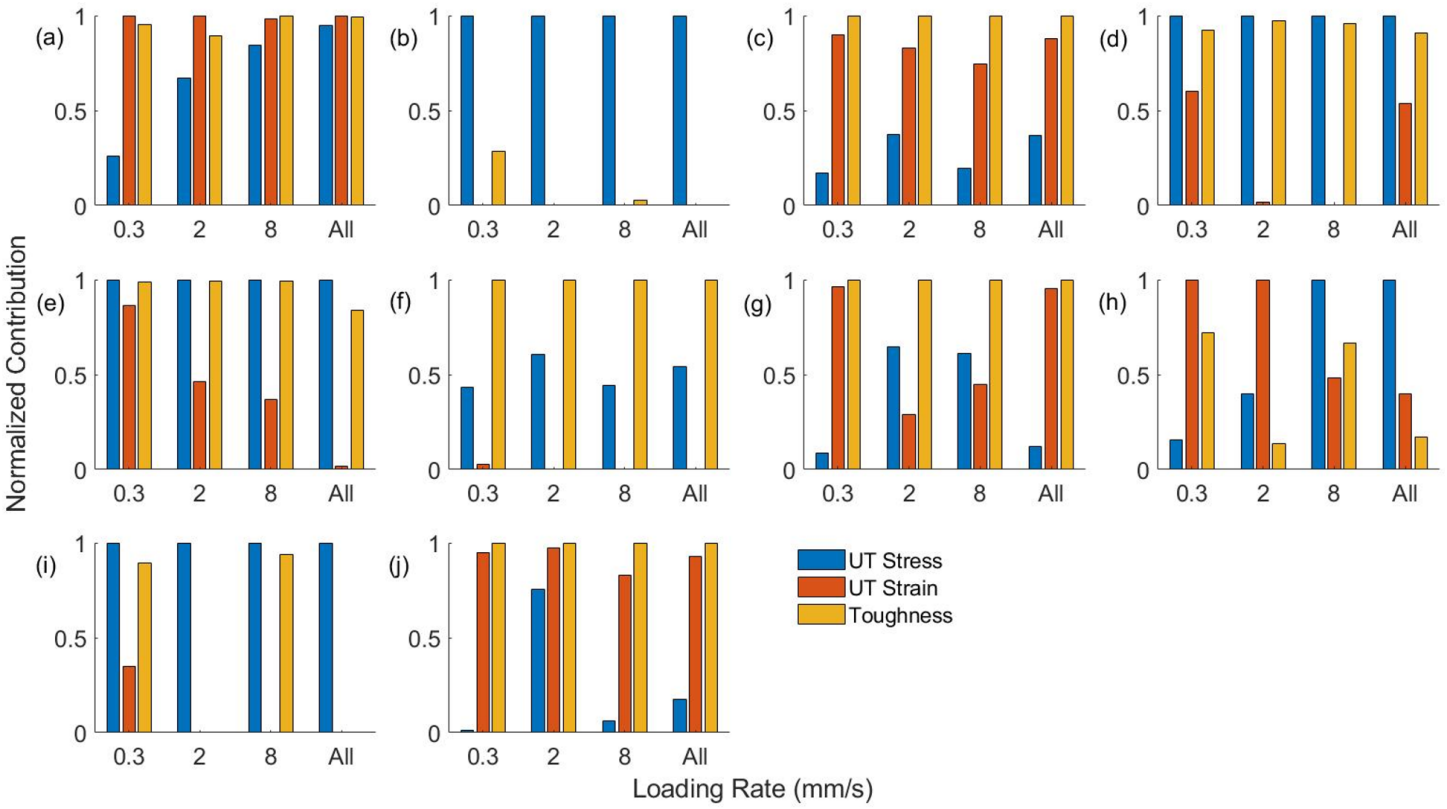
Contribution of each of the three material properties in the binary classification of full thickness burned porcine skin tissue and each of the 10 synthetic tissues ((**a**)–(**j**): tissue groups 1–10) using samples loaded at each of the three loading rates and all samples together.

To understand the contributions of each of the three mechanical properties in separating synthetics from burned porcine skin, a factor analysis of the classifier is performed. The contribution of the three properties is given in Fig. 4. All three mechanical properties are equally important since they contribute significantly to the accuracy of classifying at least one synthetic tissue group and the porcine skin tissue. UT stress contributes significantly to the accuracy of classification of porcine skin tissue and synthetic tissue groups 2, 4, 5, 8, and 9. The contribution of UT strain is prominent for classification of porcine skin tissue and synthetic tissue groups, 1, 3, 7, and 10. Toughness has significant contribution towards the accuracy in classification of the porcine tissue and synthetic tissue groups, 1, 3, 4, 5, 6, 7, and 10. Hence, all three mechanical properties studied in the present work indicate differences between the synthetic tissue groups and the burned porcine skin tissue.

## Conclusion

In this work, the fidelity of synthetic tissues used in physical simulators is evaluated by characterizing their mechanical properties under different loading rates and comparing them with those of ex vivo full thickness burned porcine skin tissues. The samples were tested at three different rates relevant to skin surgery, i.e., 0.3 mm/s, 2 mm/s, and 8 mm/s. Multivariate binary classification analysis using logistic regression and leave-one-out cross validation shows that there is significant difference between the mechanical characteristics of synthetic tissues and ex vivo burned porcine skin tissue at all loading rates. The results indicate a significant mismatch between the behavior of burned ex vivo biological tissue and that of synthetic tissues used in medical training. All of the ten synthetic tissues show a rate dependent behavior similar to the burned porcine tissue with the behavior at static loading rate distinctly different from the behavior at rates relevant to skin surgery.

A limitation of the present study is the use of ex vivo porcine tissue samples as the benchmark for comparison with synthetic tissues. Conclusive evidence of the fidelity of synthetic tissues would come from a comparison with in vivo human tissues. There are two challenges to this approach, and the obvious one is obtaining access to burned human patients. Further, uniaxial testing will not be possible for in vivo tissues. Alternatives to using in vivo human tissue include cadaveric or discarded human tissues, which may have similar limitations as the present study, *i.e.,* lack of blood perfusion, pre-stress, and muscle activation etc.^31^. Another limitation is the use of uniaxial testing in our analysis. Biaxial testing may be considered to assess anisotropic characteristics of synthetics versus burned skin tissue. It should be noted that burn care simulators need to satisfy other visual, anatomical, and physiological criteria which are beyond the scope of the present work. In spite of these limitations, it is expected that the results of this study will provide guidance for the development of more realistic high-fidelity burn care simulators that can be used as part of the Advanced Burn Life Support course.

## Supplementary Information

Supplementary Information.

## Data Availability

The datasets generated during and/or analyzed during the current study are available from the corresponding author on reasonable request.
