# Supplementary material for: Synthetic tissues lack the fidelity for the use in burn care simulators
Source: Sci Rep. 2022 Dec 10;12:21398. doi: 10.1038/s41598-022-25234-x (PMC9741590; doi:10.1038/s41598-022-25234-x)
Supplement: Supplementary file 1 — Supplementary Information. [file 41598_2022_25234_MOESM1_ESM.docx]

**Supplementary Information**

Synthetic tissues lack the fidelity for the use in burn care simulators

Vanessa Hannay^1^, Rahul*^3^, Kartik Josyula^3^, Uwe Kruger^1^, Samara Gallagher^2^, Sangrock Lee^2,3^, Hanglin Ye^2,3^, Basiel Makled^4^, Conner Parsey^4^, Jack Norfleet^4^, Suvranu De^3^

^1^Department of Biomedical Engineering, Rensselaer Polytechnic Institute, Troy NY

^2^Department of Mechanical, Aerospace, and Nuclear Engineering, Rensselaer Polytechnic Institute, Troy NY

^3^Center for Modeling, Simulation, and Imaging in Medicine, Rensselaer Polytechnic Institute, Troy NY

^4^U.S. Army Futures Command, Combat Capabilities Development Command Soldier Center STTC, Orlando FL

**Appendix A**

The mechanical behavior of each of the 10 synthetic tissues is compared with the full thickness burned porcine skin tissue using the multivariate classification analysis based on logistic regression model and leave-one-out cross validation on the three mechanical properties, i.e., ultimate tensile (UT) stress, UT strain, and toughness using samples loaded at 0.3 mm/s, 2 mm/s, 8 mm/s, and using all samples together. The confusion matrix for the classification using samples from each of the three loading rates and all samples together is given in Tables A.1 through A.4.

**Table A.1.** Confusion matrix for the classification using samples loaded at 0.3 mm/s. Note that TG refers to ‘tissue group’.

|  | Porcine | TG #1 |  |  | Porcine | TG #2 |
| --- | --- | --- | --- | --- | --- | --- |
| Porcine | 17 | 0 |  | Porcine | 17 | 0 |
| TG #1 | 0 | 15 |  | TG #2 | 0 | 15 |
|  |  |  |  |  |  |  |
|  | Porcine | TG #3 |  |  | Porcine | TG #4 |
| Porcine | 17 | 0 |  | Porcine | 17 | 0 |
| TG #3 | 0 | 14 |  | TG #4 | 0 | 15 |
|  |  |  |  |  |  |  |
|  | Porcine | TG #5 |  |  | Porcine | TG #6 |
| Porcine | 17 | 0 |  | Porcine | 17 | 0 |
| TG #5 | 0 | 15 |  | TG #6 | 0 | 15 |
|  |  |  |  |  |  |  |
|  | Porcine | TG #7 |  |  | Porcine | TG #8 |
| Porcine | 17 | 0 |  | Porcine | 17 | 0 |
| TG #7 | 0 | 15 |  | TG #8 | 0 | 15 |
|  |  |  |  |  |  |  |
|  | Porcine | TG #9 |  |  | Porcine | TG #10 |
| Porcine | 17 | 0 |  | Porcine | 17 | 0 |
| TG #9 | 0 | 15 |  | TG #10 | 0 | 15 |

**Table A.2.** Confusion matrix for the classification using samples loaded at 2 mm/s. Note that TG refers to ‘tissue group’.

|  | Porcine | TG #1 |  |  | Porcine | TG #2 |
| --- | --- | --- | --- | --- | --- | --- |
| Porcine | 24 | 0 |  | Porcine | 24 | 0 |
| TG #1 | 0 | 15 |  | TG #2 | 0 | 15 |
|  |  |  |  |  |  |  |
|  | Porcine | TG #3 |  |  | Porcine | TG #4 |
| Porcine | 24 | 0 |  | Porcine | 24 | 0 |
| TG #3 | 0 | 15 |  | TG #4 | 0 | 15 |
|  |  |  |  |  |  |  |
|  | Porcine | TG #5 |  |  | Porcine | TG #6 |
| Porcine | 24 | 0 |  | Porcine | 24 | 0 |
| TG #5 | 0 | 14 |  | TG #6 | 0 | 15 |
|  |  |  |  |  |  |  |
|  | Porcine | TG #7 |  |  | Porcine | TG #8 |
| Porcine | 24 | 0 |  | Porcine | 24 | 0 |
| TG #7 | 0 | 15 |  | TG #8 | 0 | 15 |
|  |  |  |  |  |  |  |
|  | Porcine | TG #9 |  |  | Porcine | TG #10 |
| Porcine | 24 | 0 |  | Porcine | 24 | 0 |
| TG #9 | 0 | 15 |  | TG #10 | 0 | 15 |

**Table A.3.** Confusion matrix for the classification using samples loaded at 8 mm/s. Note that TG refers to ‘tissue group’.

|  | Porcine | TG #1 |  |  | Porcine | TG #2 |
| --- | --- | --- | --- | --- | --- | --- |
| Porcine | 10 | 0 |  | Porcine | 10 | 0 |
| TG #1 | 0 | 15 |  | TG #2 | 0 | 15 |
|  |  |  |  |  |  |  |
|  | Porcine | TG #3 |  |  | Porcine | TG #4 |
| Porcine | 10 | 0 |  | Porcine | 10 | 0 |
| TG #3 | 0 | 15 |  | TG #4 | 0 | 14 |
|  |  |  |  |  |  |  |
|  | Porcine | TG #5 |  |  | Porcine | TG #6 |
| Porcine | 10 | 0 |  | Porcine | 10 | 0 |
| TG #5 | 0 | 15 |  | TG #6 | 0 | 15 |
|  |  |  |  |  |  |  |
|  | Porcine | TG #7 |  |  | Porcine | TG #8 |
| Porcine | 10 | 0 |  | Porcine | 10 | 0 |
| TG #7 | 0 | 15 |  | TG #8 | 0 | 14 |
|  |  |  |  |  |  |  |
|  | Porcine | TG #9 |  |  | Porcine | TG #10 |
| Porcine | 10 | 0 |  | Porcine | 10 | 0 |
| TG #9 | 0 | 15 |  | TG #10 | 0 | 13 |

**Table A.4.** Confusion matrix for the classification using samples loaded at all three rates. Note that TG refers to ‘tissue group’.

|  | Porcine | TG #1 |  |  | Porcine | TG #2 |
| --- | --- | --- | --- | --- | --- | --- |
| Porcine | 51 | 0 |  | Porcine | 51 | 0 |
| TG #1 | 0 | 45 |  | TG #2 | 0 | 45 |
|  |  |  |  |  |  |  |
|  | Porcine | TG #3 |  |  | Porcine | TG #4 |
| Porcine | 51 | 0 |  | Porcine | 51 | 0 |
| TG #3 | 0 | 44 |  | TG #4 | 0 | 44 |
|  |  |  |  |  |  |  |
|  | Porcine | TG #5 |  |  | Porcine | TG #6 |
| Porcine | 50 | 1 |  | Porcine | 51 | 0 |
| TG #5 | 0 | 44 |  | TG #6 | 0 | 45 |
|  |  |  |  |  |  |  |
|  | Porcine | TG #7 |  |  | Porcine | TG #8 |
| Porcine | 51 | 0 |  | Porcine | 51 | 0 |
| TG #7 | 1 | 44 |  | TG #8 | 0 | 44 |
|  |  |  |  |  |  |  |
|  | Porcine | TG #9 |  |  | Porcine | TG #10 |
| Porcine | 51 | 0 |  | Porcine | 51 | 0 |
| TG #9 | 0 | 45 |  | TG #10 | 0 | 43 |

The rate dependent behavior of each of the full thickness burned porcine skin tissue and the 10 synthetic tissues is analyzed using the multivariate classification analysis based on logistic regression model and kernel Fisher discriminant analysis (kFDA) along with leave-one-out cross validation on the three mechanical properties, i.e., ultimate tensile (UT) stress, UT strain, and toughness. The samples of each tissue type are classified into the three loading rates of 0.3 mm/s, 2 mm/s, and 8 mm/s. The confusion matrix for the multiclass classification of samples from each tissue type using logistic regression model and kFDA is given in Table A.5 and A.6, respectively. The confusion matrix for the binary classification of samples from each tissue type into static and surgical loading rates using logistic regression model is given in Table A.7.

**Table A.5.** Confusion matrix for the classification of samples from each tissue type into three loading rate groups using logistic regression model. Note that TG refers to ‘tissue group’.

| Porcine | 0.3 mm/s | 2 mm/s | 8 |  | TG #1 | 0.3 mm/s | 2 mm/s | 8 mm/s |
| --- | --- | --- | --- | --- | --- | --- | --- | --- |
| 0.3 mm/s | 12 | 5 | 0 |  | 0.3 mm/s | 10 | 2 | 3 |
| 2 mm/s | 4 | 19 | 1 |  | 2 mm/s | 0 | 12 | 3 |
| 8 mm/s | 2 | 8 | 0 |  | 8 mm/s | 4 | 5 | 6 |
|  |  |  |  |  |  |  |  |  |
| TG #2 | 0.3 mm/s | 2 mm/s | 8 mm/s |  | TG #3 | 0.3 mm/s | 2 mm/s | 8 mm/s |
| 0.3 mm/s | 15 | 0 | 0 |  | 0.3 mm/s | 14 | 0 | 0 |
| 2 mm/s | 0 | 10 | 5 |  | 2 mm/s | 0 | 11 | 4 |
| 8 mm/s | 0 | 5 | 10 |  | 8 mm/s | 0 | 1 | 14 |
|  |  |  |  |  |  |  |  |  |
| TG #4 | 0.3 mm/s | 2 mm/s | 8 mm/s |  | TG #5 | 0.3 mm/s | 2 mm/s | 8 mm/s |
| 0.3 mm/s | 13 | 2 | 0 |  | 0.3 mm/s | 14 | 1 | 0 |
| 2 mm/s | 2 | 12 | 1 |  | 2 mm/s | 0 | 13 | 1 |
| 8 mm/s | 0 | 1 | 13 |  | 8 mm/s | 0 | 0 | 15 |
|  |  |  |  |  |  |  |  |  |
| TG #6 | 0.3 mm/s | 2 mm/s | 8 mm/s |  | TG #7 | 0.3 mm/s | 2 mm/s | 8 mm/s |
| 0.3 mm/s | 9 | 6 | 0 |  | 0.3 mm/s | 13 | 1 | 1 |
| 2 mm/s | 9 | 4 | 2 |  | 2 mm/s | 0 | 14 | 1 |
| 8 mm/s | 0 | 0 | 15 |  | 8 mm/s | 5 | 4 | 6 |
|  |  |  |  |  |  |  |  |  |
| TG #8 | 0.3 mm/s | 2 mm/s | 8 mm/s |  | TG #9 | 0.3 mm/s | 2 mm/s | 8 mm/s |
| 0.3 mm/s | 15 | 0 | 0 |  | 0.3 mm/s | 15 | 0 | 0 |
| 2 mm/s | 0 | 13 | 2 |  | 2 mm/s | 0 | 11 | 4 |
| 8 mm/s | 0 | 4 | 10 |  | 8 mm/s | 0 | 0 | 15 |
|  |  |  |  |  |  |  |  |  |
| TG #10 | 0.3 mm/s | 2 mm/s | 8 mm/s |  |  |  |  |  |
| 0.3 mm/s | 11 | 3 | 1 |  |  |  |  |  |
| 2 mm/s | 3 | 12 | 0 |  |  |  |  |  |
| 8 mm/s | 2 | 0 | 11 |  |  |  |  |  |

**Table A.6.** Confusion matrix for the classification of samples from each tissue type into three loading rate groups using kFDA. Note that TG refers to ‘tissue group’.

| Porcine | 0.3 mm/s | 2 mm/s | 8 mm/s |  | TG #1 | 0.3 mm/s | 2 mm/s | 8 mm/s |
| --- | --- | --- | --- | --- | --- | --- | --- | --- |
| 0.3 mm/s | 11 | 6 | 0 |  | 0.3 mm/s | 10 | 2 | 3 |
| 2 mm/s | 0 | 24 | 0 |  | 2 mm/s | 0 | 14 | 1 |
| 8 mm/s | 0 | 9 | 1 |  | 8 mm/s | 3 | 7 | 5 |
|  |  |  |  |  |  |  |  |  |
| TG #2 | 0.3 mm/s | 2 mm/s | 8 mm/s |  | TG #3 | 0.3 mm/s | 2 mm/s | 8 mm/s |
| 0.3 mm/s | 15 | 0 | 0 |  | 0.3 mm/s | 12 | 0 | 2 |
| 2 mm/s | 0 | 12 | 3 |  | 2 mm/s | 0 | 11 | 4 |
| 8 mm/s | 0 | 3 | 12 |  | 8 mm/s | 0 | 0 | 15 |
|  |  |  |  |  |  |  |  |  |
| TG #4 | 0.3 mm/s | 2 mm/s | 8 mm/s |  | TG #5 | 0.3 mm/s | 2 mm/s | 8 mm/s |
| 0.3 mm/s | 12 | 3 | 0 |  | 0.3 mm/s | 12 | 3 | 0 |
| 2 mm/s | 1 | 14 | 0 |  | 2 mm/s | 0 | 14 | 0 |
| 8 mm/s | 0 | 7 | 7 |  | 8 mm/s | 0 | 2 | 13 |
|  |  |  |  |  |  |  |  |  |
| TG #6 | 0.3 mm/s | 2 mm/s | 8 mm/s |  | TG #7 | 0.3 mm/s | 2 mm/s | 8 mm/s |
| 0.3 mm/s | 15 | 0 | 0 |  | 0.3 mm/s | 9 | 1 | 5 |
| 2 mm/s | 9 | 5 | 1 |  | 2 mm/s | 0 | 15 | 0 |
| 8 mm/s | 0 | 6 | 9 |  | 8 mm/s | 3 | 6 | 6 |
|  |  |  |  |  |  |  |  |  |
| TG #8 | 0.3 mm/s | 2 mm/s | 8 mm/s |  | TG #9 | 0.3 mm/s | 2 mm/s | 8 mm/s |
| 0.3 mm/s | 15 | 0 | 0 |  | 0.3 mm/s | 15 | 0 | 0 |
| 2 mm/s | 6 | 9 | 0 |  | 2 mm/s | 0 | 11 | 4 |
| 8 mm/s | 4 | 6 | 4 |  | 8 mm/s | 0 | 0 | 15 |
|  |  |  |  |  |  |  |  |  |
| TG #10 | 0.3 mm/s | 2 mm/s | 8 mm/s |  |  |  |  |  |
| 0.3 mm/s | 14 | 0 | 1 |  |  |  |  |  |
| 2 mm/s | 1 | 14 | 0 |  |  |  |  |  |
| 8 mm/s | 2 | 0 | 11 |  |  |  |  |  |

**Table A.7.** Confusion matrix for the binary classification of samples from each tissue type into static and surgical rate groups using logistic regression model. Note that TG refers to ‘tissue group’.

| Porcine | Static | Surgical |  | TG #1 | Static | Surgical |
| --- | --- | --- | --- | --- | --- | --- |
| Static | 12 | 5 |  | Static | 11 | 4 |
| Surgical | 4 | 30 |  | Surgical | 4 | 26 |
|  |  |  |  |  |  |  |
| TG #2 | Static | Surgical |  | TG #3 | Static | Surgical |
| Static | 15 | 0 |  | Static | 14 | 0 |
| Surgical | 0 | 30 |  | Surgical | 0 | 30 |
|  |  |  |  |  |  |  |
| TG #4 | Static | Surgical |  | TG #5 | Static | Surgical |
| Static | 13 | 2 |  | Static | 14 | 1 |
| Surgical | 2 | 27 |  | Surgical | 0 | 29 |
|  |  |  |  |  |  |  |
| TG #6 | Static | Surgical |  | TG #7 | Static | Surgical |
| Static | 8 | 7 |  | Static | 10 | 5 |
| Surgical | 8 | 22 |  | Surgical | 4 | 26 |
|  |  |  |  |  |  |  |
| TG #8 | Static | Surgical |  | TG #9 | Static | Surgical |
| Static | 10 | 5 |  | Static | 10 | 5 |
| Surgical | 4 | 26 |  | Surgical | 4 | 26 |
|  |  |  |  |  |  |  |
| TG #10 | Static | Surgical |  |  |  |  |
| Static | 6 | 9 |  |  |  |  |
| Surgical | 6 | 22 |  |  |  |  |
